# Warmer temperature accelerates methane emissions from the Zoige wetland on the Tibetan Plateau without changing methanogenic community composition

**DOI:** 10.1038/srep11616

**Published:** 2015-06-25

**Authors:** Mengmeng Cui, Anzhou Ma, Hongyan Qi, Xuliang Zhuang, Guoqiang Zhuang, Guohui Zhao

**Affiliations:** 1 Key Laboratory of Environmental Biotechnology, Research Center for Eco-Environmental Sciences, Chinese Academy of Sciences, Beijing 100085, China; 2 The Georgia State University, 50 Decatur St SE, Atlanta, GA 30303

## Abstract

Zoige wetland, locating on the Tibet Plateau, accounts for 6.2% of organic carbon storage in China. However, the fate of the organic carbon storage in the Zoige wetland remains poorly understood despite the Tibetan Plateau is very sensitive to global climate change. As methane is an important greenhouse gas and methanogenesis is the terminal step in the decomposition of organic matter, understanding how methane emissions from the Zoige wetland is fundamental to elucidate the carbon cycle in alpine wetlands responding to global warming. In this study, microcosms were performed to investigate the effects of temperature and vegetation on methane emissions and microbial processes in the Zoige wetland soil. A positive correlation was observed between temperature and methane emissions. However, temperature had no effect on the main methanogenic pathway—acetotrophic methanogenesis. Moreover, methanogenic community composition was not related to temperature, but was associated with vegetation, which was also involved in methane emissions. Taken together, these results indicate temperature increases methane emissions in alpine wetlands, while vegetation contributes significantly to methanogenic community composition and is associated with methane emissions. These findings suggest that in alpine wetlands temperature and vegetation act together to affect methane emissions, which furthers a global warming feedback loop.

Methane (CH_4_) is the second most abundant greenhouse gas (GHG) after carbon dioxide (CO_2_)^1^, and accounts for 14 percent of GHG emissions^2^. Despite the fact that CH_4_ is emitted into the atmosphere in smaller quantities than CO_2_, its global warming potential (i.e., the ability of the gas to trap heat in the atmosphere) is 25 times greater. Wetlands are the largest source of atmospheric CH_4_ emissions, accounting for approximately 170 Tg CH_4_ per year from natural wetlands and 39–112 Tg CH_4_ per year from constructed wetlands. Climate change is predicted to affect CH_4_ emissions from natural wetlands through multiple avenues. For example, temperature^3^,^4^,^5^, and vegetation^6^ all play important roles in CH_4_ emissions. CH_4_ emission fluctuations from natural wetlands, caused by climate changes, may further contribute to global warming.

Of particular concern is the impact of global warming on CH_4_ emissions from permafrost wetlands^7^, which contribute significantly to carbon sequestration. CH_4_ emission levels from wetlands are strongly correlated with soil temperature. For example, CH_4_ emissions exhibited strong temperature-dependence, with higher CH_4_ emissions at higher temperatures in studies of different peatlands^8^,^9^. As the largest highland wetland in the world, the Zoige wetland is located in the northeast corner of the Qinghai-Tibet Plateau^10^. The average height of the Tibet Plateau is 4000 meters above sea level, known colloquially as “the roof of the world”. Besides the Arctic and Antarctic, the Tibet Plateau is often called “the third pole of the earth”, which makes it a key area for studying global changes^11^. In the past fifty years, there has been a rise in temperatures of up to 0.3 °C a decade in the Tibet Plateau— approximately three times the global warming rate^11^. Obviously, the Tibet Plateau is very sensitive to global climate change. Comprised primarily of peat bogs, the Zoige wetland area is the largest peat deposition in China. Its carbon storage is estimated to be 5.54 Pg, accounting for 6.2% of the organic carbon storage in China^10^. With climate change, there is potential for the Zoige wetland to release its stored carbon as additional CO_2_ and CH_4_ into the atmosphere, forming a positive feedback loop through increases in greenhouse emissions. In this manner, it may accelerate global warming, contributing to a negative cycle of global climate change.

In addition to temperature, vegetation also plays an important role in CH_4_ emissions from wetlands. Vegetation not only provides a conduit for CH_4_ emissions by way of aerenchyma but also provides substrates for CH_4_ production by means of root decay and exudation^12^. CH_4_ emissions have been found to be positively correlated with vegetation biomass in the Qinghai-Tibetan Plateau wetland^13^ in addition to several other wetland ecosystems^6^,^14^. In contrast, other studies have shown a negative correlation between CH_4_ emissions and vegetation biomass^15^,^16^. Additionally, it has been observed that vegetation community composition is another important factor influencing CH_4_ emissions from wetlands, with distinct rates of CH_4_ emissions from wetlands dominated by different plants^15^,^16^. Climate change can also have a profound effect on vegetation community composition^17^. Changes in this community composition in response to global warming may further alter CH_4_ emission from wetlands.

CH_4_ production (methanogenesis) is the terminal step during microbial decomposition of organic matter (OM) in anaerobic environments (natural wetlands, lake sediments and flooded rice fields), and is performed by methanogens^18^. There are two main types of methanogenic pathways: acetate- and H_2_/CO_2_-dependent methanogenesis^19^. Acetotrophic methanogenesis is considered the dominant pathway, and normally contributes twice as much to total CH_4_ production as the alternative hydrogenotrophic methanogenesis pathway. However, the relative contributions of the two pathways may vary under different conditions. Fey *et al*. showed that hydrogenotrophic methanogenesis is the predominant pathway of CH_4_ formation under thermophilic conditions^20^,^21^. It was also found that CH_4_ production occurs mainly from H_2_/CO_2_ in peat, especially in deeper layers, accounting for 50 to 100% of the total production amounts^22^,^23^. On the other hand, the percent of CH_4_ production from acetate is greater than 80% in an ombrotrophic bog in Michigan^3^. The relative contribution of each pathway is crucial for studying methane emissions. As methanogenesis by CO_2_ reduction exhibits a much stronger fractionation factor than acetoclastic methanogenesis^24^, we can use values of δ^13^C measured in CH_4_, CO_2_ and acetate to compute the relative contributions of the two pathways^25^. Several previous studies have focused on methanogenesis within the Zoige area^26^,^27^. However, the effects of global warming on methane emissions in this area have yet to be fully elucidated.

This study provides insight into the potential consequences of global warming on CH_4_ emissions by investigating changes in carbon mineralization at ambient temperature (15 °C) and the predicted temperature in 2100 (20 °C)^2^. Specially, this work examines variation in primary methanogenic pathways and in methanogenic community structures of the Zoige wetland. As a typical highland peatland, Zoige wetland harbor a special plant species community, which is mainly constituted by the plant species *Carex muliensis* (C), *Eleocharis valleculosa* (E) and *Polygonum amphibium* (P)^28^. Moreover, *Carex muliensis* and *Eleocharis valleculosa* cover about 95% of the entire site^29^. In an attempt to isolate the effect of temperature, all incubations were performed under uniform anaerobic conditions.

## Results

### Carbon mineralization (CH_4_ and CO_2_ production)

**Figure 1:**
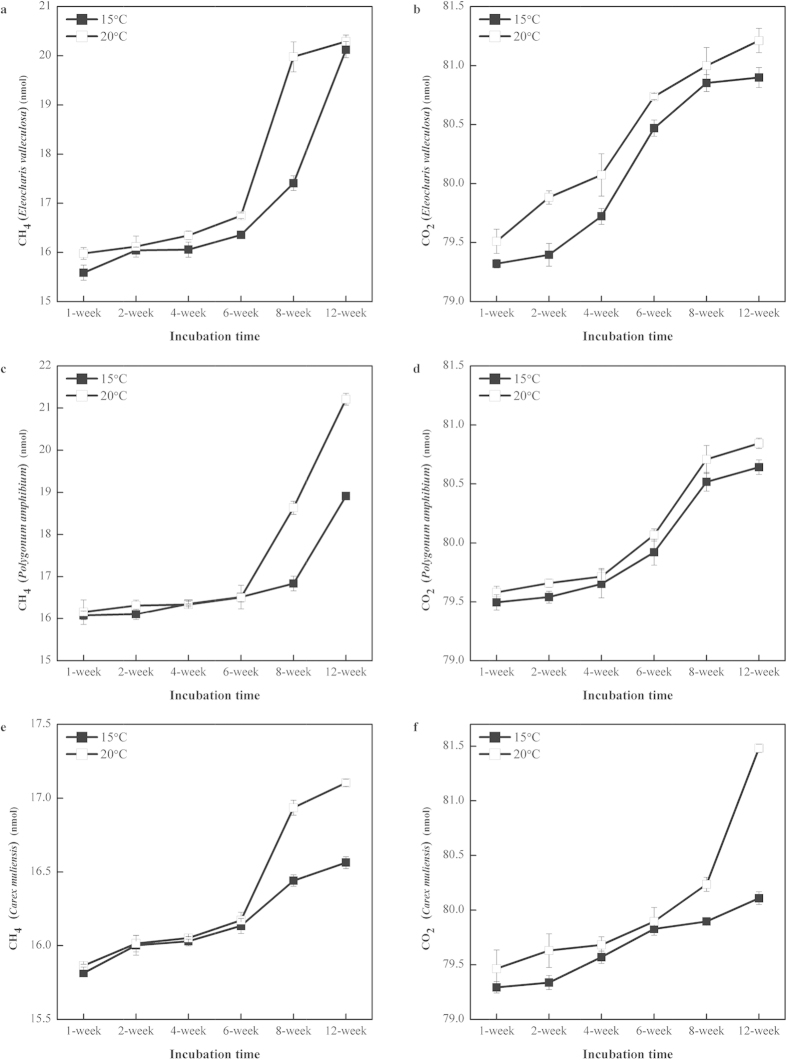
Time course of accumulation of CH_4_ and CO_2_ in incubation at 15 and 20 °C using samples from three kinds of plant-dominated soil in Zoige wetland, i.e., *Eleocharis valleculosa* (E), *Polygonum amphibium* (P), *Carex muliensis* (C); mean ± SE_mean_, n = 3.

Accumulation of CH_4_ and CO_2_ was lower at 15 °C than at 20 °C (Fig. 1a–f). CO_2_ concentration increased during the whole incubation time, ranged from 79 nmol to 82 nmol (Fig. 1b,d,f). Two phases of CH_4_ dynamics could be distinguished (Fig. 1a,c,e). During the first phase (the first six weeks) CH_4_ production remained slow. In the second stage (weeks 6–12) CH_4_ production shifted into a more active phase, which corresponded with a rapid decrease in the acetate concentration (Supplementary Fig. S1b, S1d, S1f). At week 8 the concentration of CH_4_ was significantly [one-way ANOVA F test: P = 1.75 × 10^−4^ for C (*Carex muliensis*); P = 1.97 × 10^−4^ for E (*Eleocharis valleculosa*); and P = 1.86 × 10^−4^ for P (*Polygonum amphibium*)] increased with the 5 °C temperature increase for all three types of incubations (Fig. 1a,c,e). CH_4_ concentrations in C soil incubations were lower than the other two kinds of incubations E and P throughout the 12 weeks (Fig. 1a,c,e).

### Stable carbon isotope signatures of CH_4_ and CO_2_

**Figure 2:**
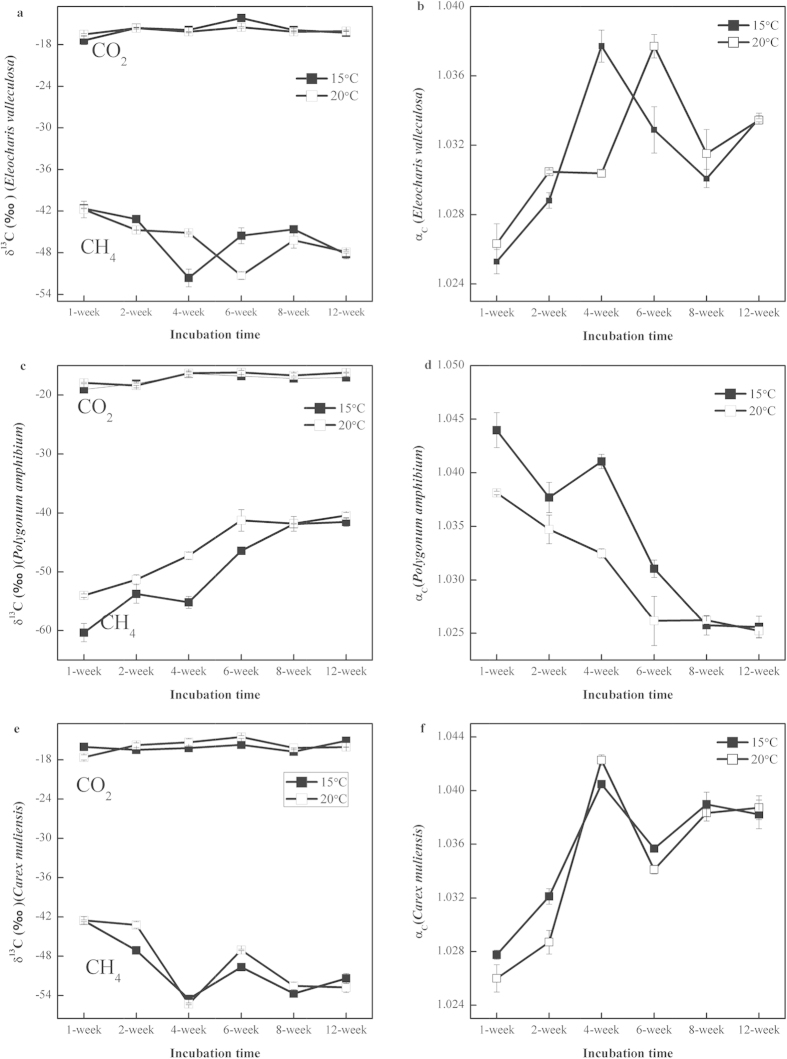
Evolution of δ^13^C values of accumulated CH_4_ and CO_2_, and apparent fractionation factors α_c_ in incubation at 15 and 20 °C using samples from three kinds of plant-dominated soil in Zoige wetland, i.e., *Eleocharis valleculosa*, *Polygonum amphibium*, *Carex muliensis*; mean ± SE_mean_, n = 3.

The values of δ^13^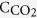 in all incubations showed a similar trend and remained almost solely in the range from −20‰ to −14‰ (Fig. 2a,c,e). The values of δ^13^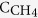 in P soil incubations increased from −60‰ to −41‰ (Fig. 2c), which differed from the other two types of incubations E (Fig. 2a) and C (Fig. 2e). For E and C incubations, the values of δ^13^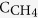 initially decreased during the slow CH_4_ production period to reach their lowest values −52‰ and −54‰, respectively (Fig. 2a,e). The values of δ^13^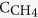 then increased to reach their highest values, −41‰ for E (Fig. 2a) and −42‰ for C (Fig. 2e), at week 8 and week 6, respectively. Finally, the values of δ^13^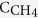 decreased again (Fig. 2a,e). Moreover, the values of δ^13^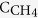 were higher at 20 °C than at 15 °C in almost all samples (Fig. 2a,c,e). The apparent fractionation factor α_c_ ranged from 1.025 to 1.033 for E (Fig. 2b), from 1.025 to 1.043 for P (Fig. 2d), and from 1.025 to 1.049 for C (Fig. 2f).

### T-RFLP analysis of archaeal and bacterial communities

**Figure 3:**
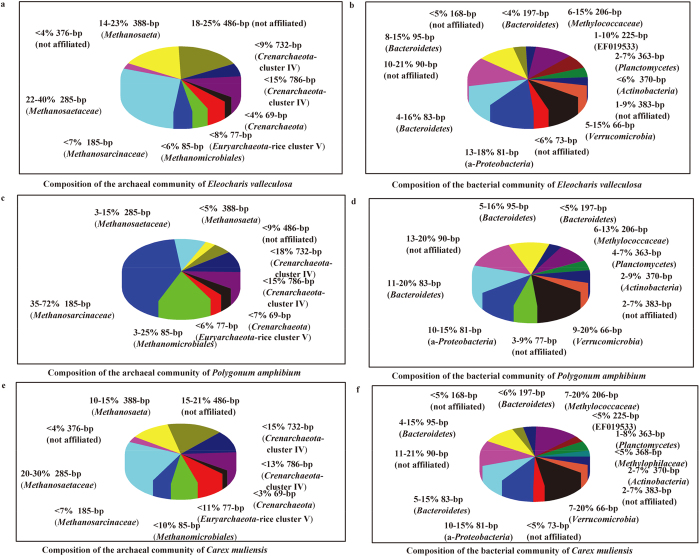
Relative abundance of individual T-RFs from T-RFLP analysis targeting archaeal and bacterial 16S rRNA genes in incubation using samples from three kinds of plant-dominated soil in Zoige wetland, i.e., *Eleocharis valleculosa*, *Polygonum amphibium*, *Carex muliensis*. The data shown in this figure are consensus of all datasets, which were obtained from all the six sampling time points (week 1, week 2, week 4, week 6, week 8 and week 12) during the incubation period.

The composition of the methanogenic community was determined by archaeal 16 S rRNA gene-based T-RFLP. The results of the T-RFLP analyses showed characteristic differences in the methanogenic communities at diverse conditions (Fig. 3a,c,e). The methanogenic patterns in P incubations were different from those in E and C incubations (Fig. 3a,c,e). However, the structures of the methanogenic communities in all incubations remained relatively constant over the entire incubation period, and were not significantly affected by the rise in temperature (Supplementary Fig. S2).

Compared to *Archaea*, the composition of the bacterial community was much more complex, as revealed by bacterial 16S rRNA gene-based T-RFLP (Fig. 3) Bacterial communities were not significantly influenced by incubation time or the rise in temperature (Supplementary Fig. S3). As with the archaeal pattern, the bacterial pattern was strongly correlated with vegetation type.

### Sequencing and quality control

A total of 15 samples were taken from three types of incubations. These samples were analyzed by Illumina sequencing of the 16S rRNA V4 region. The number of clean reads was 1,126,858, the average number of reads was 75,123 and the average length was 252 bp.

### Alpha (α) diversity indices

To compare the diversity indices, we normalized the sequence number of each sample to 38,200 reads. Tags with 97% similarity were then grouped into OTUs (Operational Taxonomic Units) to calculate the rarefaction curves and diversity indices (Supplementary Table S1 and Fig. S4a).

The Shannon-Wiener diversity index considers both richness and evenness. Rarefaction curves of the Shannon index were different from those of the observed species, as they approached a plateau from less than 10,000 tags per sample (Supplementary Fig. S4b). Moreover, the temperature rise had almost no effect on the diversity of microbes (Supplementary Fig. S4 and Table S1).

### Archaeal community analysis: taxonomy composition, clustering and beta (β) diversity

For all samples, bacteria were the most abundant microbial group (95.9%–99.1%), followed by archaea (0.8%–4%) and “not assigned” (about 0.1%). The overall microbial community was separated into archaeal assemblage and bacterial assemblage, neither of which were significantly affected by the temperature rise.

**Figure 4:**
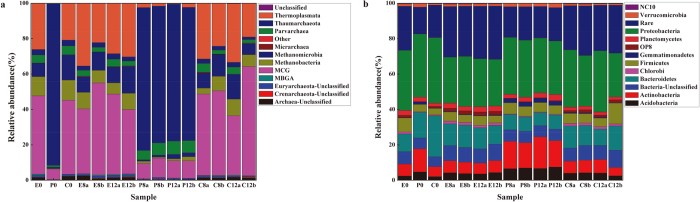
Distribution of phylogenetic groups: Archaea (**a**) and Bacteria (**b**). (**a**) Relative abundance of the dominant archaeal classes in the three kinds of incubations. (**b**) Relative abundance of the dominant bacterial phyla in the three kinds of incubations.

In archaeal assemblage, *Thaumarchaeota* (75%–92%) was the major group and the two methanogenic archaeal classes (*Methanobacteria* and *Methanomicrobia*) represented only 1% to 4.6% of the total archaeal sequences of P group (Fig. 4a), which was different from those in the E and C groups. While the pattern of archaea distribution in group E showed that MCG (36%–52%) and *Thermoplasmata* (22%–36%) were the dominant groups, the two methanogenic archaeal classes comprised about 15%–20% of the total archaeal sequences (Fig. 4a). Group C’s archaea distribution shared the same pattern as E, however the relative abundance were slightly different from group E. In group C, the relative abundance of MCG, *Thermoplasmata* and the two methanogenic archaeal classes (*Methanobacteria* and *Methanomicrobia*) were 33%–62%, 19%–35%, and 12%–26%, respectively (Fig. 4a).

Based on the OTU-level data, sample clustering produced more intuitive resolution for differentiating the archaeal communities, and most groups within incubation samples clustered together with relatively high similarity (Supplementary Fig. S5a). The clustering results also indicated that groups of E and C incubation communities were more similar to each other than to the P incubation. This result coincided well with the TRFLP analysis. Moreover, a species classification clustering (Supplementary Fig. S5a) showed that the dominant species in E incubations were identical to C while both were different from P incubations, demonstrating that archaeal communities were selected by their habitats.

### Bacterial community analysis: taxonomy composition, clustering and beta (β) diversity

For bacterial distribution, the three incubation groups shared a similar pattern (Fig. 4b). The top 10 most abundant phyla were: *Proteobacteria*, *Bacteroidetes*, *Actinobacteria*, *Firmicutes*, *Acidobacteria*, *Planctomycetes*, *Verrucomicrobia*, *Chlorobi*, *Gemmatimonadetes* and OP8. However, the specific proportion of each phylum differed slightly among the three types of incubations (Supplementary Table S2). The pattern of bacterial distribution (Fig. 4b) was almost entirely consistent with the TRFLP analysis of bacterial communities.

For bacterial communities, the clustering analysis (Supplementary Fig. S5b) showed groups E and C samples clustering together with higher similarity to each other than to P incubations, consistent with TRFLP results as well as the trend observed in the distribution of archaeal communities.

## Discussion

The aim of this study was to examine changes in CH_4_ emissions and methanogenic community structure along a temperature gradient from the ambient temperature to the predicted temperature for year 2100 to better understand how temperature affects CH_4_ emissions in permafrost wetland systems. The role of temperature as an influencing factor on CH_4_ emissions from wetlands is particularly important in light of the potential for climate change-associated global warming. There have been few studies that focus on the effects of global warming on CH_4_ emissions from the Zoige wetland, even though it is the largest highland wetland in the world and is located on the Tibet Plateau^10^, an ideal place for global warming study due to its susceptibleness to climate change^11^.

The positive relationship between CH_4_ emissions and temperature exhibited in the current work is consistent with previous studies, which demonstrates that CH_4_ emissions are strongly related to temperature, with higher CH_4_ emissions at higher temperatures in different peatlands^8^,^30^ and ecosystems^31^. We consider two nonexclusive possible explanations for this pattern: (i) decreased vegetation abundance and/ or altered community composition, which changes CH_4_ emissions by influencing CH_4_ production and transport; and (ii) different methanogen compositions at different habitats.

Different rates of CH_4_ emissions have been observed from wetlands dominated by different plants, which demonstrates that the vegetation community composition also plays an important role in CH_4_ emissions^15^,^16^. Our study showed a similar pattern, with lower rates of CH_4_ emissions from C incubations than from E and P incubations, which suggested that these plant species led to changes in CH_4_ emissions in the Zoige wetland. There is no report mentioning effects of climate warming on abundances of the above three plant species internationally. However, it has been found that plant species diversity has declined dramatically and rapidly with climate warming in Tibet Plateau ecosystems^32^. CH_4_ emissions were suggested positive associated with plant species diversity^14^. Moreover, the results of partial correlation analysis showed that CH_4_ emissions were significantly related to vegetation compared to other site specific factors, such as organic matter and pH (Supplementary Table S3). Thus, the main types of vegetation in the Zoige wetland might manipulate the CH_4_ emissions from soil.

The second explanation of the temperature-CH_4_ emissions relationship we observed is the effect of temperature on methanogens^33^. There are two main types of methanogenic pathways: acetate- and H_2_/CO_2_-dependent methanogenesis^19^, which are performed by different methanogens^18^. In our study we observed that the first six week period of CH_4_ production (Fig. 1a) constituted a lag phase before CH_4_ accumulation. After sampling events, the soils were stored at −20 °C until use in experiments, which led to the perturbation of samples. So eventual recovery needed a period resulting in such a long lag phase, as previously reported for peat^34^ and rice soil^21^. This was consistent with acetate dynamics (Supplementary Fig. S1), as acetate is considered to be the main substrate of methanogenesis^19^.

The 5 °C rise in temperature had no effects on the main methanogenesis processes, suggested by the apparent fractionation factors in this study. However, the fluctuated values of δ^13^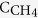 showed that the relative importance of the two pathways changed during the incubation. This is because CH_4_ production by acetoclastic methanogenesis exhibits a much lower fractionation factor (α_c_ < 1.055) than hydrogenotrophic methanogenesis (α_c_ > 1.065)^25^. Most of the incubations at 20 °C showed larger δ^13^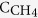 values than at 15 °C, demonstrating that the temperature rise enhanced the activity of acetoclastic methanogenesis compared to hydrogenotrophic methanogenesis. For E and C groups, the relative proportion of hydrogenotrophic methanogenesis increased as δ^13^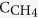 values declined. The following increase in δ^13^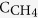 values suggested that acetoclastic methanogenesis became more and more active. A similar δ^13^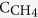 trend was reported by Qu^35^. On the other hand, δ^13^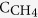 increased continuously, demonstrating that the relative importance of acetoclastic methanogenesis increased throughout the entire P incubation, accompanied by decreased activity of hydrogenotrophic methanogenesis. This was different from the patterns observed in E and C incubations. This was consistent with TRFLP results and illumina-based sequencing analyses showing that the methanogenic community composition of group P was different from groups E and C. It has been indicated that archaeal community composition is related to vegetation type^36^. A wide range of labile carbon compounds including organic acids, sugars, phenolics and amino acids released into the soil might stimulate methanogens and their activities^15^.

Our finding that both acetate and H_2_/CO_2_ served as methanogenic precursors was in accordance with our results on methanogenic community composition. Based on Illumina sequencing data, the dominant methanogenic community *Methanosarcinales* most likely accounted for acetoclastic methanogenesis and *Methanobacteriales* for hydrogenotrophic methanogenesis^37^. *Methanosarcinales* are mostly acetoclastic methanogens but are also able to use H_2_, methanol, trimethylamine and other C1 compounds^38^. As a common community of methanogens, *Methanosarcinales* were detected in various locations, such as an acidic West-Siberian peat bog^38^, flooded Italian rice fields^20^, and high Arctic peat^34^. Known members of *Methanobacteriales* grow exclusively through the CO_2_-reduction pathway, using one or more of the substrates H_2_/CO_2_, formate and short-chained alcohols^4^. In a previous study by Metje and Frenzel^4^, *Methanobacteriales* were the only type of methanogens in an acidic peat of Northern Finland and hydrogenotrophic methanogenesis was the dominant methanogenic pathway.

In contrast, there have been some minor methanogenic communities revealed, such as *Methanomicrobiales* for hydrogenotrophic methanogenesis^37^, which were detected in two American peatlands and in an acidic West-Siberian peat bog^39^. Our results differed slightly from those of Zhang^26^, indicating that members of *Methanosarcinales* and *Methanomicrobiales* constituted the majority of methanogens in the Zoige wetland, while *Methanobacteriales* were present at low ratios. This difference may be caused by the fact that *Methanomicrobiales* can be cold selective hydrogenotrophic methanogens. In addition, Rice cluster II (RC- II) were detected in our incubations. These are most likely methanogens due to their phylogenetic placement close to *Methanosarcinales* and *Methanomicrobiales*^40^. However, a recent study by Conrad^41^ showed that RC- II are most likely not methanogenic, based on the fact that the *mcrA* tree (representing methanogenic archaea only) does not show a separate cluster that would be homologous to that of RC- II on the 16 S rRNA gene tree. The methanogenic phenotype of RC- II requires further study.

The patterns of Illumina sequencing in all three types of incubations shown in Fig. 4a did not indicate any temperature-dependent changes in the structure of archaeal communities. However, the CH_4_ concentrations were improved with a 5 °C rise in all three types of incubations (Fig. 1a). This may be due to enhanced activity of methanogens despite their consistent quantities (Supplementary Fig. S2).

*Proteobacteria* and *Bacteroidetes* dominated most bacterial communities indicating that the dominant groups were *Proteobacteria* (51.6%) and *Bacteroidetes* (17.7%) in the Zoige Alpine Wetland. As a member of *Proteobacteria*, aerobic methanotrophs are responsible for CH_4_ oxidation, which is an important part of CH_4_ cycle^42^,^43^,^44^. Two methanotrophic families, including *Methylococcaceae* (type I) and *Methylocystaceae* (type II), were detected in the Zoige wetland (Supplementary Table S4), and the methanotrophs’ relative abundances were close to each other among the three kinds of incubations. *Bacteroidetes* seem to specialize in the digestion of other polysaccharides^45^. *Firmicutes* were also contributors to the bacterial assemblage. These have been shown to be relevant in fermentation of organic matter in anoxic rice field soil, in particular during the methanogenic degradation of straw and plant residues^46^ and appear to be the main degraders of cellulose^45^, which could be assessed by stable-isotope probing, a powerful and widely used technique to identify active microorganisms involved in specific metabolic processes^47^,^48^.

In summary, temperature was found to be positively correlated with CH_4_ emissions from the largest highland wetland in the world — the Zoige wetland (Fig. 1a). Despite this correlation, the dominant pathway (acetoclastic methanogenesis) and methanogen community composition were not influenced by temperature. However, the methanogen community composition was related to vegetation type, with different patterns at different habitats (Fig. 3 and Supplementary Fig. S5). The results of our study suggest that temperature and vegetation act together to influence CH_4_ emissions from wetlands. At the same time vegetation is also affected by climate warming^32^ and in turn influences CH_4_ emissions, which seems as self-regulation in response to global warming. More research on how vegetation changes with global warming is needed if we are to quantitatively predict global warming-induced changes in CH_4_ emissions from wetlands and attempt to highlight the putative underlying mechanisms of self-regulation as global warming progresses.

## Methods

### Sites and sampling

Our study sites in the Zoige National Wetland Reserve (33°56′ N, 102°52′ E) are located at the eastern edge of the Tibetan Plateau in southwestern China. From June to September soil temperatures are between 6 °C and 15 °C^26^. The mean annual rainfall is approximately 650 mm. The peat remained water saturated in appearance at all times and was not affected by water table fluctuations. *Eleocharis valleculosa* (E), *Polygonum amphibium* (P) and *Carex muliensis*(C) are the three dominant plants in these wetlands and the peat soils associated with these plants were selected for our study. The depth of standing water remained in a range from 5 to 15 cm in the peat over the sampling period. Soils were sampled in early August 2011 because water and soil in marshes are completely frozen from late October to the following April, begin to melt in late April, and the highest temperatures occur in July. In order to minimize disturbance to the natural environment and facilitate sampling, three sampling plots at each marsh were subjectively scattered along the boardwalk. The distance of the adjacent marsh was about 100 meters. The size of an individual plot was 10 cm^3^ below water. The fresh soil was immediately stored at 4 °C and transported to a laboratory for use in soil property measurements (Table 1). The soils were then stored at −20 °C until use in experiments.

### Incubation experiments

The incubation procedure was designed according to studies by Keller^49^. The soil samples were diluted 1:1(vol/vol) with anoxic sterile water and then blended. Approximately 40-mL aliquots were placed into 100-mL sterile test bottles. The bottles were closed with butyl rubber stoppers, flushed with N_2_ and incubated at either 15 or 20 °C for 12 weeks. Three parallel slurries were made for each temperature, plant soil, and sampling time point. The sampling time points were week 1, week 2, week 4, week 6, week 8 and week 12. There were three types of samples to be analyzed at each sampling point, including gas, liquid, and sediment. Before sampling the headspace gas, bottles were shaken vigorously to allow equilibration between the liquid and gas phases. Gas samples were analyzed for both concentrations and δ^13^C values of CH_4_ and CO_2_. The slurries were centrifuged at 17,949 g at 4 °C for 15 min to separate the supernatants from the sediments^50^. The supernatants were used to measure pH values and acetate concentrations, while the sediments were stored at −20 °C for nucleic acid extraction.

### Analytical techniques

The concentrations of CH_4_ and CO_2_ in the headspace gas were measured using a Shimadzu (2010 ultra) gas chromatograph with an Agilent GS-CarbonPLOT column (30 m × 0.32 mm × 1.5 μm) (Agilent Technologies, USA) and a mass spectrometry detector (GC-MS) with He as the carrier gas. Injection, detection and column temperatures were 150, 200 and 35 °C, respectively^51^. Liquid samples were filtered with 0.22-μm hydrophilic polyethersulfone (PES) syringe filters (Shanghai Anpel, China) and stored at −20 °C until analysis. pH values were measured using a Mettler Toledo pH meter (FE20 Plus, Shanghai). Concentrations of acetate in the liquid phase of the soil incubations were analyzed by a Shimadzu (2010 ultra) gas chromatograph with an Agilent DB-FFAP column (30 m × 0.25 mm × 0.25 μm) (Agilent Technologies, USA) and a flame ionization detector, with N_2_ as the carrier gas. Thawed samples were acidified by the addition of 6 g/L formic acid to retain the molecular morphology of the volatile acid. Injection, detection and column temperatures were 200, 200 and 125 °C, respectively. The isotopic composition (δ^13^C values) of CH_4_ and CO_2_ were determined using a Deltav Advantage gas chromatograph combustion isotope ratio mass spectrometer system (GCC-IRMS) (Thermo scientific, USA)^21^. The analytical procedures were the same as for GC-MS. The apparent isotopic fractionation factor was determined by α_app_ = (δ^13^CO_2_ + 10^3^)/(δ^13^CH_4_ + 10^3^)^25^.

### DNA extraction and PCR amplification

DNA was extracted using a FastDNA SPIN kit for soil (MP, Germany) according to the manufacturer’s instructions. Archaeal 16S rRNA was amplified using the primer combination Ar109f/Ar915r^52^, with the reverse primer labeled with 6-carboxyfluorescein (FAM) (Life Technologies, China). The PCR was performed as follows: 45 s at 94 °C, 45 s at 52 °C, and 90 s at 72 °C for 30 cycles, with a primary denaturation step of 5 min at 94 °C and final DNA synthesis for 10 min at 72 °C. Bacterial 16 S rRNA were amplified using primers 27 F/1392 R^53^, with the forward primer labeled with 6-carboxyfluorescein (FAM) (Life Technologies, China). The PCR was performed as follows: 60 s at 94 °C, 60 s at 52 °C, and 90 s at 72 °C for 30 cycles, with a primary denaturation step of 5 min at 94 °C and final DNA synthesis for 10 min at 72 °C. PCR products were purified with an E.Z.N.A.™ Cycle-Pure Kit (Omega, USA).

### T-RFLP analysis

T-RFLP was performed as described previously^54^. After purification using an E.Z.N.A.™ Cycle-Pure Kit (Omega, USA), the PCR products of archaeal DNA were digested at 65 °C for 3 h by *Taq*I (Thermo scientific, USA). Bacterial PCR products were digested at 37 °C for 3 h by *Hha*I (NEB, USA). The digestion products were sent to Tsingke (Beijing, China) for detection. The assignment of T-RFs to taxonomic groups was performed by MiCA, which is accessible at http://mica.ibest.uidaho.edu/55.

### Illumina sequencing of 16S rRNA V4 region

Based on the results of carbon mineralization, all three types of DNA incubation samples at 0-week, 8-week and 12-week time points were sequenced as follows:

### DNA sample preparation for sequencing (Novogene Experimental Department)

16S rRNA V4 region, including both bacterial and archaeal communities, were amplified using the specific primers (515F/806R) with the barcode. All PCR reactions were carried out in 30 μL reactions with 15 μL of Phusion^®^ High-Fidelity PCR Master Mix (NEB, USA); 0.2 μM of forward and reverse primers, and about 10 ng template DNA. Thermal cycling consisted of initial denaturation at 98 °C for 1 min, followed by 30 cycles of denaturation at 98 °C for 10 s, annealing at 50 °C for 30 s, and elongation at 72 °C for 30 s. Finally 72 °C for 5 min. A total amount of 200 ng amplicon per sample was used as input material for the DNA sample preparation. Sequencing libraries were generated using the Illumina Truseq™ DNA Sample Preparation Kit (Illumina, San Diego, USA) following the manufacturer’s recommendations and 15 index codes were used to identify different samples. Remaining overhangs were converted into blunt ends via exonuclease/polymerase activities and enzymes were removed. After adenylation of 3′ ends of DNA fragments, Illumina PE adapter oligonucleotides were ligated to prepare samples for hybridization. In order to preferentially choose DNA fragments of 291 bp in length, agrose electrophoresis was performed (120 V, 40 min, 1.5% agarose gel) and adapter-ligated constructs from 250 bp to 350 bp were isolated. After purification using a spin column (QIAGEN, Dusseldorf, Germany), DNA fragments with ligated adapter molecules on both ends were selectively enriched using the Illumina PCR Primer Cocktail in a 10 cycle PCR reaction. Products were purified using the AMPure XP system (Beckman Coulter, Beverly, USA) and quantified using the Agilent high sensitivity DNA assay on the Agilent Bioanalyzer 2100 system.

### Clustering and sequencing

Clustering of the index-coded samples was performed on a cBot Cluster Generation System using a TruSeq PE Cluster Kit v3-cBot-HS (Illumina, San Diego, USA) according to the manufacturer’s instructions. After cluster generation, library preparations were sequenced on an Illumina Miseq platform and 250 bp paired-end reads were generated.

### Data analysis

Pairs of reads from the original DNA fragments were merged by using FLASH^56^, a very fast and accurate software tool which is designed to merge pairs of reads when the original DNA fragments are shorter than twice the length of reads. Sequencing reads were assigned to each sample according to the unique barcode of each sample.

After removing all singletons from datasets, sequences were analyzed with the QIIME^57^ software package (Quantitative Insights Into Microbial Ecology), in addition to custom Perl scripts to analyze alpha (within sample) and beta (between sample) diversity. First the reads were filtered using QIIME quality filters. We then used “pick_de_novo_otus.py” to pick operational taxonomic units (OTUs) through creation of an OTU table. Sequences were assigned to OTUs at 97% similarity. We chose a representative sequence for each OTU and used the RDP classifier^58^ to assign taxonomic data to each representative sequence. In order to compute Alpha Diversity, we rarified the OTU table and calculated three metrics; the Chao1 metric which estimates species richness, the Observed Species metric which is simply the count of unique OTUs found in the sample, and Shannon-Wiener diversity index. Rarefaction curves were generated based on these three metrics.

QIIME calculates both weighted and unweighted unifrac, which are phylogenetically aware measures of beta diversity. We used unweighted unifrac to do an Unweighted Pair Group Method with Arithmetic mean (UPGMA) Clustering. UPGMA Clustering is a type of hierarchical clustering using average linkage and can be used to interpret the distance matrix.

## Additional Information

**How to cite this article**: Cui, M. *et al*. Warmer temperature accelerates methane emissions from the Zoige wetland on the Tibetan Plateau without changing methanogenic community composition. *Sci. Rep*. **5**, 11616; doi: 10.1038/srep11616 (2015).

## Supplementary Material

Supplementary Information

## Figures and Tables

**Table 1:** Soil characteristics of different sampling sites in this experiment.

Site	Characteristic						
	Moisture^a^ (%)	pH^b^	Organic matter^c^ (g/Kg)	Total N^d^ (g/Kg)	NH_4_^+^-N^e^ (mg/Kg)	NO_3_^—^N^e^ (mg/Kg)	Total P^f^ (g/Kg)
*Eleocharis valleculosa*	89.74 ± 2.51	7.10 ± 0.12	91.62 ± 0.13	1.728 ± 0.0031	21.06 ± 0.037	0.0125 ± 0.0017	0.4913 ± 0.0028
*Polygonum amphibium*	63.71 ± 1.38	7.45 ± 0.015	74.68 ± 0.58	1.689 ± 0.0027	8.798 ± 0.040	0.0325 ± 0.0021	0.7026 ± 0.0071
*Carex muliensis*	77.77 ± 2.41	7.08 ± 0.11	102.2 ± 0.79	1.680 ± 0.0074	16.53 ± 0.015	0.1025 ± 0.013	0.4471 ± 0.0051

Analysis was performed at the Laboratory of Soil and Fertilizer Institute, Chinese Academy of Agricultural Sciences.

Mean ± SE_mean_, n = 3.

^a^Measured by drying at 105 °C overnight and weighing.

^b^Measured by mixing wet weight soil with distilled water at a ratio of 1:1(w/w).

^c^Determined by external heating-potassium dichromate volumetric method.

^d^Determined by the Kjeldahl method.

^e^Determined by a flow analyzer.

^f^Measured by perchloric acid-concentrated sulfuric acid digestion- molybdenum, antimony anti colorimetry.
