# Supplementary material for: Warmer temperature accelerates methane emissions from the Zoige wetland on the Tibetan Plateau without changing methanogenic community composition
Source: Sci Rep. 2015 Jun 25;5:11616. doi: 10.1038/srep11616 (PMC4479872; doi:10.1038/srep11616)

Warmer temperature accelerates methane emissions from the Zoige wetland on the Tibetan Plateau without changing methanogenic community composition

Mengmeng Cui, Anzhou Ma*, Hongyan Qi, Xuliang Zhuang, Guoqiang Zhuang*, Guohui Zhao

Supplementary information

Surprisingly, the NC10 phylum was still observed in Fig.4b despite its low abundance (0.4% of E, 0.1% of P, 0.3% of C). Moreover the NC10 phylum continuously existed in the three groups (E, P and C) of all the three sampling time points (0-week, 8-week and 12-week), and the specific abundance of NC10 in the three groups almost remained constantly as the other dominant phyla of bacteria. Because anaerobic methane oxidation coupled to denitrification (N-DAMO) was recently assigned to bacteria belonging to the uncultured NC10 phylum[1], which may play a role in CH_4 emissions from the Zoige wetland. Similarly, a previous study also detected *pmoA* (the alpha subunit of particulate methane monooxygenase sequence) from "*Candidatus* Methylomirabilis oxyfera" (*M. oxyfera*) – a member of the uncultured NC10 phylum in the Zoige wetland[2].

1. Ettwig, K. F. *et al.* Enrichment and molecular detection of denitrifying methanotrophic bacteria of the NC10 phylum. *Appl. Environ. Microb.* **75**, 3656–3662 (2009).

2. Luesken, F. A. *et al. pmoA* primers for detection of anaerobic methanotrophs. *Appl. Environ. Microb.* **77**, 3877–3880 (2011).

Table S1 Diversity indices for the sample of Illumina sequencing

Sample	Diversity index		
	Chao1	OTU	Shannon-Wiener
E0	77774	14255	11.3976
P0	89301	15954	11.87978
C0	72863	13212	10.37275
E8a	92211	15471	11.73357
E8b	86825.96	15331	11.67432
E12a	89112	15340	11.77361
E12b	101785	16708	12.09625
P8a	79997	15552	12.12393
P8b	75790	15131	12.08339
P12a	80168	15257	12.02257
P12b	80156	15507	12.14794
C8a	87972	15111	11.70994
C8b	87525	15536	11.78964
C12a	98415	16011	11.97175
C12b	84781	16015	12.0003

Eleocharis valleculosa, *Polygonum amphibium*, *Carex muliensis*; 0,0-week; 8,8-week; 12,12-week; a, 15 °C; b, 20 °C.

Table S2 The proportion of the top 10 most abundant bacterial phylum shared by the three kinds of incubation groups: *Eleocharis valleculosa* (E), *Polygonum amphibium* (P) and *Carex muliensis* (C).

Group	Bacterial phylum (%)		
	E	P	C
Proteobacteria	26-38	30-36	24-38
Bacteroidetes	10-13	7-15	9-24
Actinobacteria	6	15	5
Firmicutes	4-7	4-7	3-12
Acidobacteria	2-5	4-8	2-5
Planctomycetes	2.5	3	2
Verrucomicrobia	1	2	1
Chlorobi	15	0.4	1
Gemmatimonadetes	0.3	2	0.5
OP8	1.5	0.2	1

Table S3 The partial correlation analysis of the site specific factors affecting CH_4 emissions in the Zoige wetland by SPSS.

Correlations with CH_4 emissions [a]	15 °C		20 °C	
	Correlation	Significance (2-tailed)	Correlation	Significance (2-tailed)
Vegetation	0.959	$<10^{-4}$ **	0.989	$<10^{-4}$ **
OM [b]	0.280	0.466	0.438	0.238
pH	$<10^{-4}$	1	0.109	0.780
Moisture	0.516	0.155	0.381	0.312
NH_4^+-N	0.443	0.232	0.299	0.43
Total-P	0.064	0.871	0.225	0.561

[a] Cell contain zero-order (Pearson) correlation

[b] OM, organic matter

**, P<0.01.

Table S4 The proportion of the two methanotrophic families in the three kinds of incubation groups: *Eleocharis valleculosa* (E), *Polygonum amphibium* (P) and *Carex muliensis* (C).

Group	Methanotrophic families (%)	
	Methylococcaceae (type I)	*Methylocystaceae* (type II)
E	1.8-2.1	0-0.1
P	1-2	0-0.1
C	0.7-1.5	0-0.1

Fig.S1 The pH values and concentrations of acetate in incubation at 15 and 20 °C using samples from three kinds of plant-dominated soil in Zoige wetlands, i.e., *Eleocharis valleculosa*, *Polygonum amphibium*, *Carex muliensis*; mean ± $SE_{mean,}$ n=3.

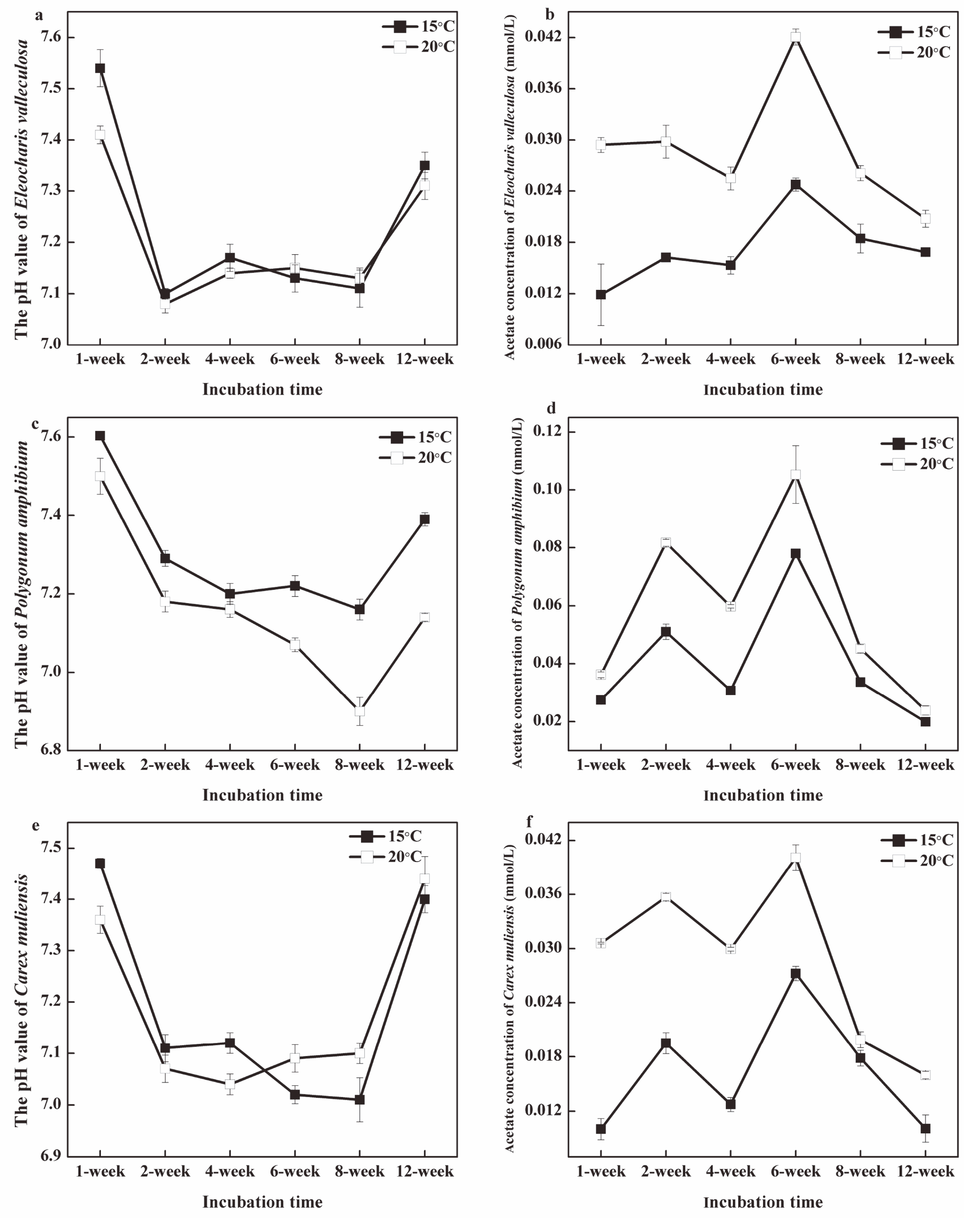

Fig.S2 Relative abundance of individual T-RFs from T-RFLP analysis targeting archaeal 16S rRNA genes in incubation at 15 and 20 °C using samples from three kinds of plant-dominated soil in Zoige wetlands, i.e., *Eleocharis valleculosa*, *Polygonum amphibium*, *Carex muliensis*; mean ± SE_{mean}, n=3.T-RFs, terminal restriction fragments; T-RFLP, terminal restriction fragment length polymorphism; Ar, archaea.

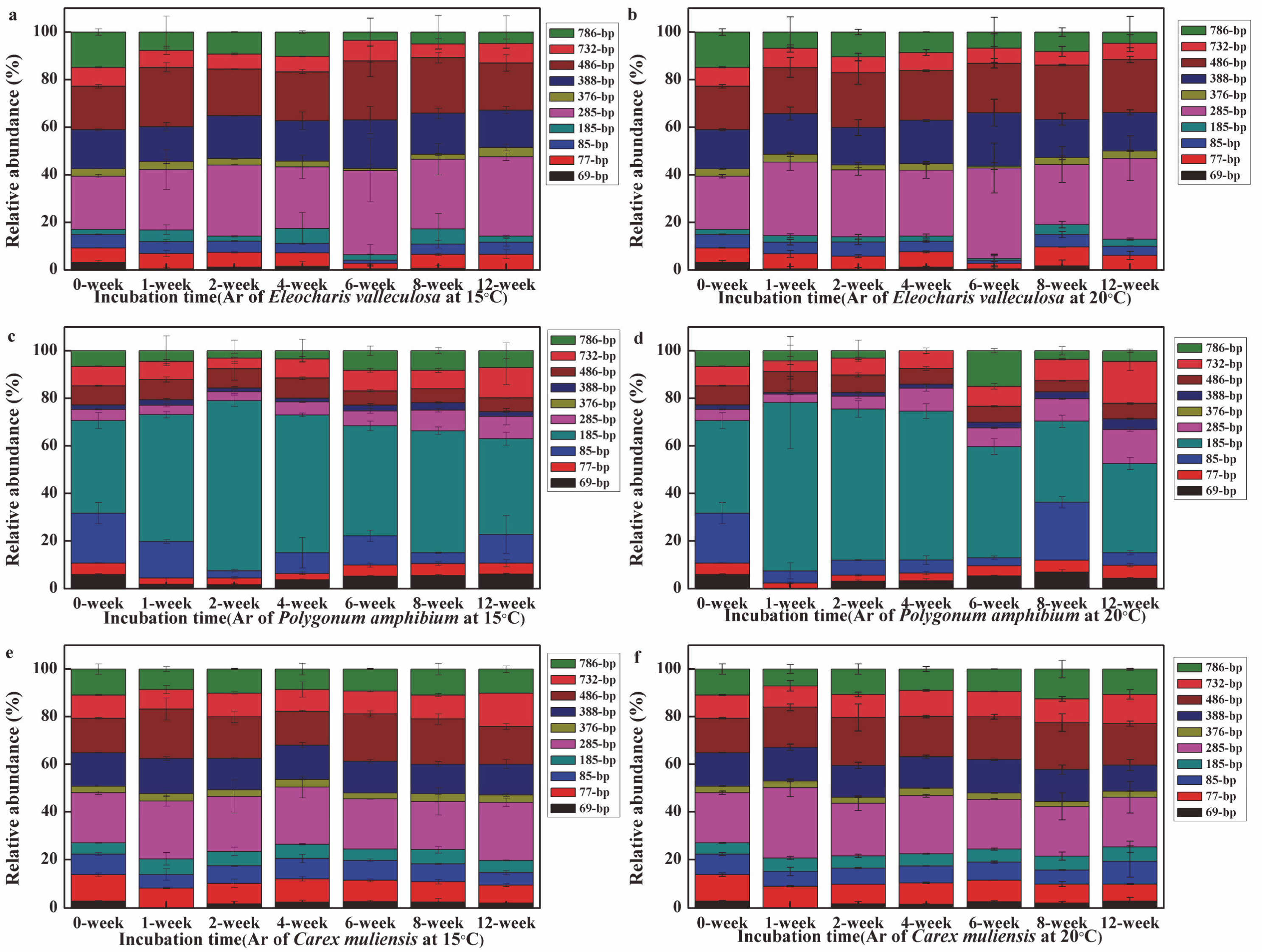

Fig.S3 Relative abundance of individual T-RFs from T-RFLP analysis targeting bacterial 16S rRNA genes in incubation at 15 and 20 °C using samples from three kinds of plant-dominated soil in Zoige wetlands, i.e., *Eleocharis valleculosa*, *Polygonum amphibium*, *Carex muliensis*; mean ± SE_{mean}, n=3.T-RFs, terminal restriction fragments; T-RFLP, terminal restriction fragment length polymorphism; Ba, bacteria.

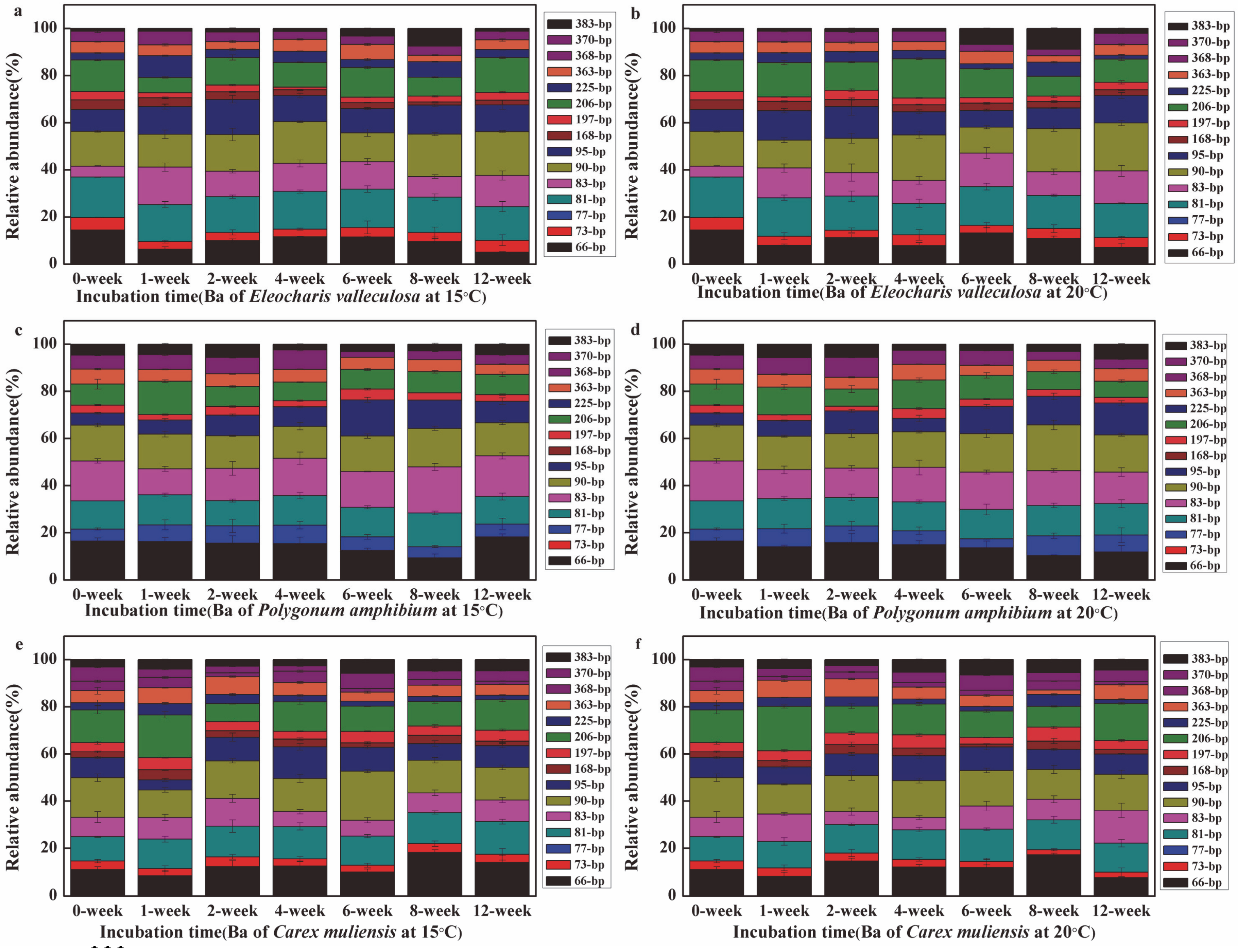

Fig.S4 α-Diversity comparison. Rarefaction curves for observed OTUs (a) and Shannon-Wiener index (b) were calculated using QIIME with reads normalized to 38,200 for each sample using 0.03 distance OTUs. *Eleocharis valleculosa*, *Polygonum amphibium*, *Carex muliensis*; 0,0-week; 8,8-week; 12,12-week; a, 15 °C; b, 20 °C.

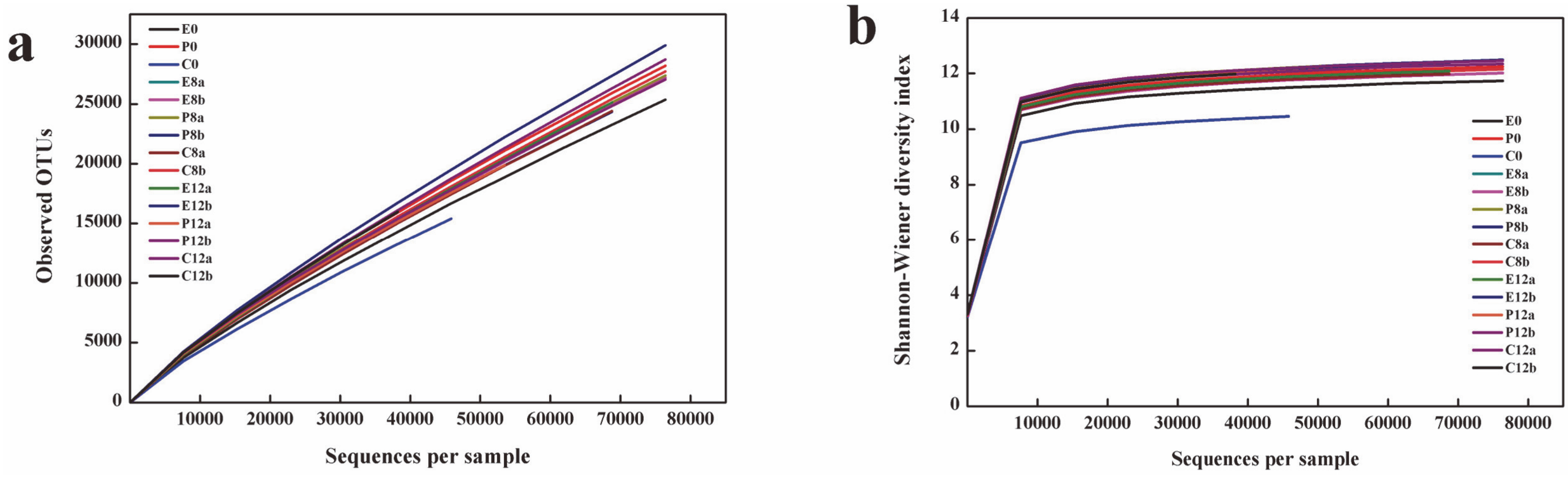

Fig.S5 Cluster analysis of OTUs: Archaea (a) and Bacteria (b), showing the status of species classification clustering (horizontal clustering) and sample clustering (vertical clustering) according to the species abundance level of each sample. Different colors meaning the degree of aggregation of species among different samples.

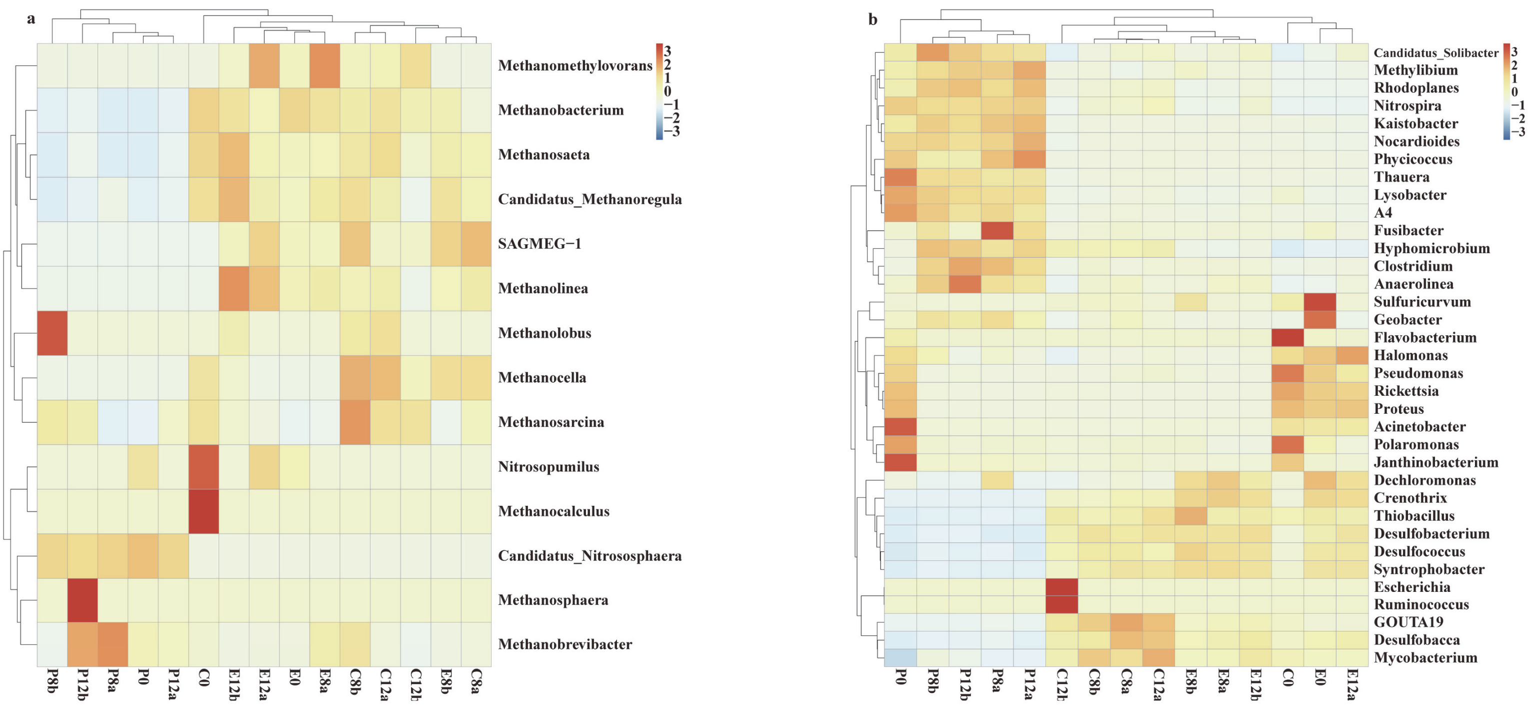

Supplement: Supplementary Information [file srep11616-s1.pdf]
